# Investigating the risk factors of penile arterial insufficiency and veno‐occlusive dysfunction in patients with erectile dysfunction

**DOI:** 10.1002/bco2.275

**Published:** 2023-08-31

**Authors:** Mohamad Habous, Ahmed Khattak, Mohammed Farag, Saleh Binsaleh, David Ralph, Mohammed Aziz, Carlo Bettocchi, Gordon Muir

**Affiliations:** ^1^ Al Themal Medical Center Abha Saudi Arabia; ^2^ King's College Hospital London UK; ^3^ Urology Department Azhar University, Assiut Branch Assiut Egypt; ^4^ Division of Urology, Department of Surgery, Faculty of Medicine King Saud University Riyadh Saudi Arabia; ^5^ St. Peters Institute of Andrology, UCLH London UK; ^6^ Urology Department Menofia University Shibin Al Kawm Egypt; ^7^ Bari University Bari Italy

**Keywords:** coronary insufficiency, erectile dysfunction, penile artery insufficiency, penile duplex, penile haemodynamics

## Abstract

**Objective:**

To investigate the risk factors for penile arterial insufficiency (PAI), which is a known cause of erectile dysfunction (ED).

**Methods:**

Patients who attended our urology clinic complaining of ED for more than 6 months were prospectively enrolled in this study over 1‐year period. Patient consent was taken and ethical committee approval. Complete medical history and thorough general and local examination including body mass index (BMI), Peyronie's disease (PD) and penile size measurements (length and girth) were done for all of them. Laboratory tests included testosterone, lipid profile and glycated haemoglobin (HA1c). A penile duplex ultrasound study (PDU) was done for all patients after intracavernosal injection (ICI) with alprostadil. Peak systolic velocity (PSV) and end‐diastolic velocity (EDV) were measured after 15 min. Statistical analysis was done using SPSS.

**Results:**

A total of 440 patients were enrolled in this analysis. The mean age was 48(23–81), and the mean BMI was 30 (18–51). Older patients had lower PSV (*r* = −0.361, *P* = 0.000) and higher EDV (*r* = 0.174, *P* = 0.001), and both correlations were highly statistically significant. Diabetics had lower PSV (*r* = −0.318, *P* = 0.000) and higher EDV (*r* = 0.139, *P* = 0.008), which were also highly statistically significant. Smokers had lower PSV (*r* = −0.140, *P* = 0.008) and higher EDV (*r* = 0.178, *P* = 0.001), which were highly statistically significant. Men with larger penises measured skin to tip had lower EDV (*r* = −0.119, *P* = 0.024), which was less significant. Interestingly, there was neither a significant correlation between BMI and PSV (0.16, *P* = 0.745) nor a significant correlation between testosterone and PSV (0.029, *P* = 0.552). Also, there was no correlation between PSV and both dyslipidaemia and penile PD.

**Conclusions:**

Ageing, tobacco consumption, DM and hypertension seem to have a negative impact on penile haemodynamics, which was statistically significant. In our patients, there was no statistically significant effect on penile haemodynamics in patients with increased BMI, low testosterone or PD or according to the size of the penis.

## 1 INTRODUCTION

Erectile dysfunction (ED) is the most frequently encountered sexual dysfunction in men worldwide. ED is defined as the recurrent or consistent inability to obtain and/or maintain an erection sufficient for satisfactory sexual intercourse.^1^ The prevalence of ED is increasing with age: It is 15% in men 40–50 years old, 45% in men 60–70 and 70% in men over 70.^2^

Several studies have demonstrated that common cardiovascular disease (CVD) risk factors are also risk factors for ED. These risk factors include age, hypertension, diabetes mellitus (DM), smoking, increased body mass index (BMI) and dyslipidaemia.^3^

Growing evidence has established that ED shares many risk factors with systemic conditions like metabolic syndrome (MTS).^4^ MTS is the most important public health issue threatening the health of men and women globally. Its current prevalence, approximately 30%, is continuously increasing. MTS by itself is considered a risk factor for ED.^5^ MTS is a combination of five cardiovascular risk factors. These risk factors include raised blood pressure (hypertension), dyslipidaemia (raised triglycerides and lowered high‐density lipoprotein cholesterol), raised fasting glucose (DM) and central obesity. Three abnormal findings out of five would qualify a person for the metabolic syndrome.^6^

Debate still exists about the association of smoking with ED. However, the evidence of such an association is likely due to the consistency of the relationship between smoking and endothelial disease and the strength of the association of ED with other endothelial diseases.^7^

The primary blood supply to the penis is through the internal pudendal artery, which arises from the anterior division of the internal iliac artery. The internal pudendal artery becomes the common penile artery after giving off a branch to the perineum. These branches are the dorsal artery, the bulbourethral artery and the cavernosal artery. The cavernosal artery is responsible for the tumescence of the corpus cavernosum, and the dorsal artery is responsible for the engorgement of the glans penis during erection. The three branches of penile arteries join distally to form a vascular ring, as an anastomosis, near the glans. The cavernosal artery gives off many helicine arteries, which supply the trabecular erectile tissue and the sinusoids. The helicine arteries are contracted and tortuous in the flaccid state and become dilated and straight during erection. Venous drainage from the three corpora (both corpora cavernosa and corpus spongiosum) originates in the tiny venules just beneath the tunica albuginea, forming the subtunical venular plexus before exiting as the emissary veins. Outside the tunica albuginea, a prominent deep dorsal vein is the main venous drainage via the circumflex vein. Then, a single deep dorsal vein runs upwards behind the symphysis pubis to join the periprostatic venous plexus.^8^

The underlying processes in vasculogenic ED are arterial insufficiency, veno‐occlusive dysfunction or combinations of both. Penile Doppler ultrasound (PDU) study is a diagnostic modality useful in elucidating the cause of ED and the magnitude of its severity.^9^ PDU also provides a dynamic, quantifiable and reliable method for evaluation of several structural conditions in the penis. It can also help in detecting fibrotic plaques and calcifications characteristic of Peyronie's disease (PD), defects in the tunica albuginea and variable echogenicity in corpora cavernosa in the case of trauma or features of priapism (differentiating between high and low flow), arteriocavernosal fistulas and high‐resistance cavernosal arterial flow in addition to anatomic variations in vasculature.^10^ The peak systolic velocity (PSV) and end‐diastolic velocity (EDV) are measured to assess penile haemodynamics. A PSV lower than 25 cm/s or an EDV greater >5 cm/s in the setting of adequate arterial flow have been the major criteria used to define and distinguish ED due to arterial insufficiency or veno‐occlusive dysfunction, respectively.^11^

We ran this study to investigate the risk factors of penile artery insufficiency.

## 2 MATERIALS AND METHODS

### 2.1 Study population

Patients who attended our urology clinic complaining of ED for more than 6 months were prospectively enrolled in this study over 1‐year period. Complete medical history including sexual history, other associated comorbidities and tobacco consumption were recorded for all patients. Thorough general and local examination including BMI, penile plaques, deformity (PD) and/or any abnormality was recorded. Laboratory tests included testosterone, lipid profile and glycated haemoglobin (HbA1c).

***Exclusion criteria*** included patients with congenital anomalies like hypospadias and congenital curvature. Patients who refused intracorporeal injection with vasoactive agents (ICI) were also excluded from this analysis. We also excluded patients with poor clinical response to ICI. Patient consent was taken and ethical committee approval.

### 2.2 ICI notes

The patients were examined in a private comfortable air‐conditioned consulting room at a constant temperature (21°C) to ensure a relaxing atmosphere. Intracavernosal Quadrimix (prostaglandin E1 5 μg, papaverine 15 mg, phentolamine 1 mg, atropine 20 mcg in 15 mL of saline). Clinical response to ICI was classified into four grades: E1: no response, E2: penile engorgement, E3: fair erection and E4: good rigid erection. Repeat dosing was done for patients with insufficient erection till at least a fair clinical response (E3). We usually use Quadrimix in ICI as its more potent than PGE to ensure the best possible clinical response is achieved, before confirming that the patient had (poor clinical response) to ICI. Patients who did not achieve fair clinical response (<E3) after ICI were excluded from this analysis. ICI was done after counselling and discussion with the patient regarding the risks and benefits of the procedure and the potential for complications including priapism.

***Penile size*** was measured at the time of full erection on the dorsal aspect of the penis, from the pubic bone to tip of the glans (BTT), from suprapubic skin to tip (STT) and circumference of base of penis.

### 2.3 PDU technique

All the procedures of colour PDU, included in this analysis, were done by a single experienced andrologist. PDU was performed using high frequency linear array ultrasound probe with the patient in the supine position. The penis was scanned from its ventral surface using longitudinal and transverse views. After an intracavernosal injection of Quadrimix, the penis was typically scanned on the ventral surface at a fixed angle. The angle between the incident beam and the vector of blood flow, that is, the angle of insinuation, was maintained at 60°.To standardise the velocity measurements (PSV and EDV), spectral sampling of the cavernosal artery was obtained at the origin, proximally on the base of the penis under the guidance of the colour Doppler signal, where the cavernosal artery angles posteriorly towards the crus. Then PSV and EDV were measured and recorded between 10 and 15 min after injection time.

***Statistical analysis*** was performed using SPSS (IBM, SPSS Statistics 21, Chicago, IL, USA). Descriptive statistics (mean, SD, median and range) were performed for age, BMI and each of the three recorded measurements of the erect penis (skin‐to‐tip length, bone‐to‐tip length and circumference). Pearson correlation coefficients were calculated using SPSS for each of the recorded variables.

## 3 RESULTS

**FIGURE 1:**
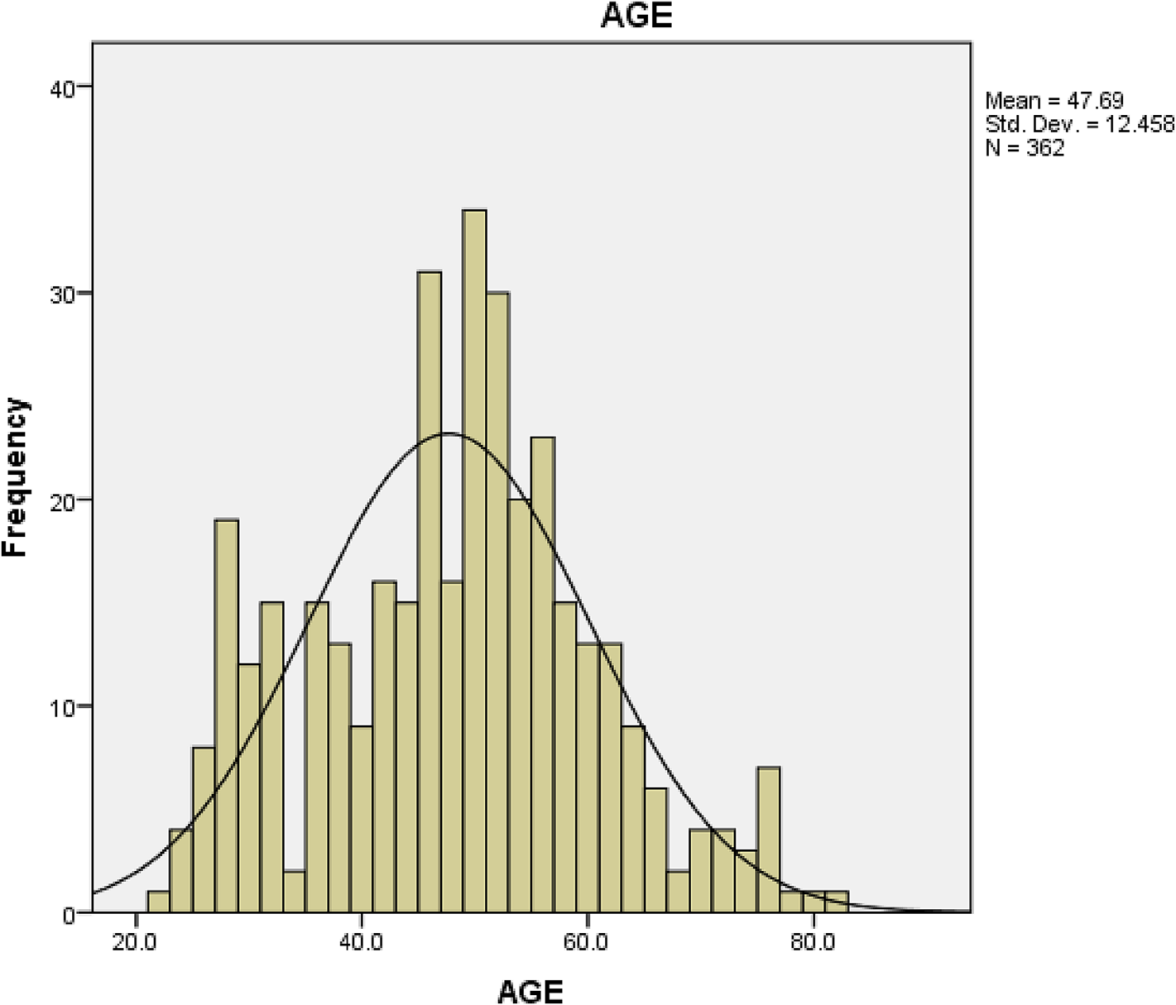
Graphical representation of age data in the cohort.

**FIGURE 2:**
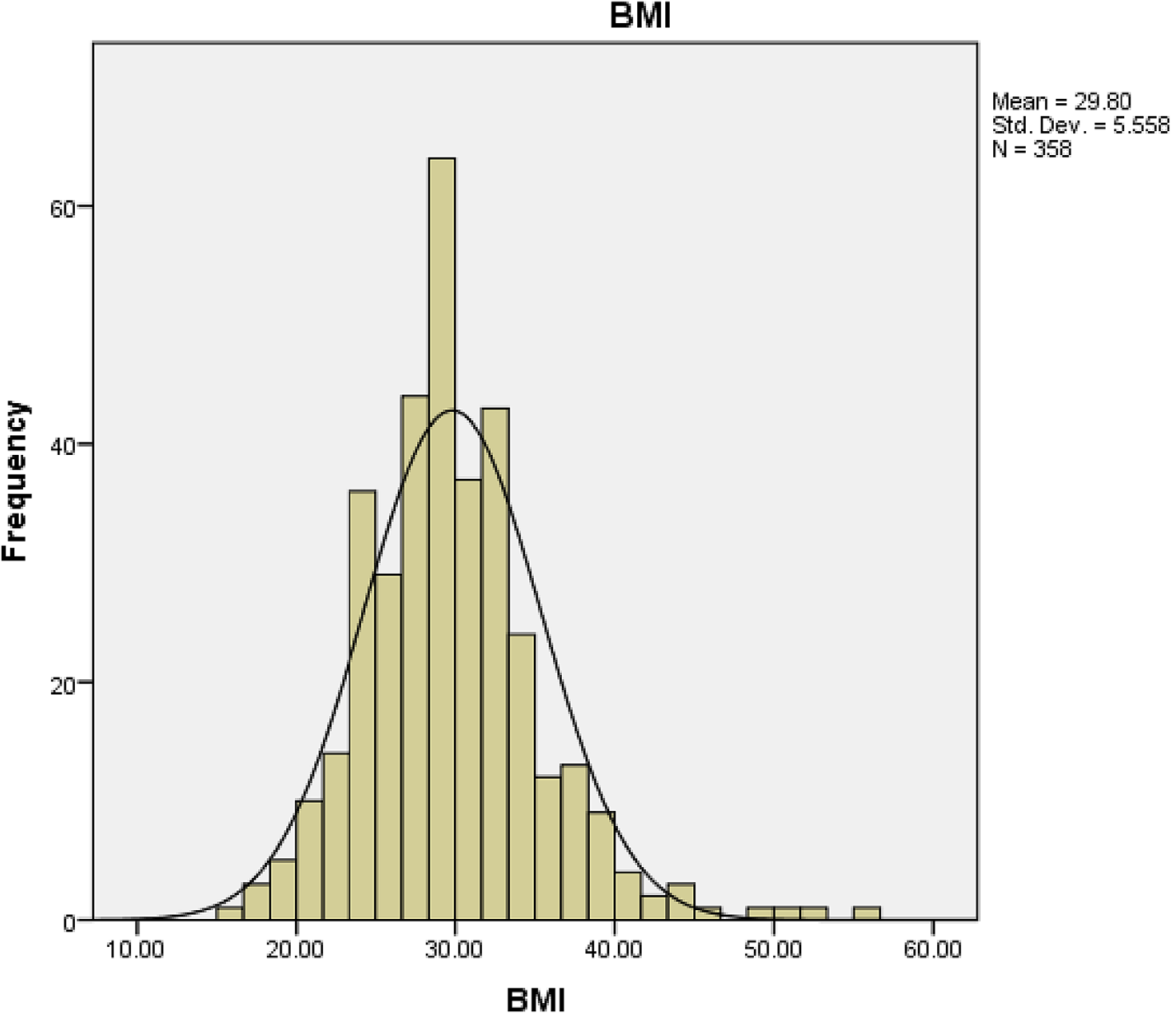
Graphical representation of BMI data among the cohort.

Four hundred and forty men were enrolled in this study, but those with complete data were only 362. The mean age was 48 (23–81); for more details about age, see Figure 1.The mean BMI was 30 (18–52); for more details about BMI, see Figure 2.

**FIGURE 3:**
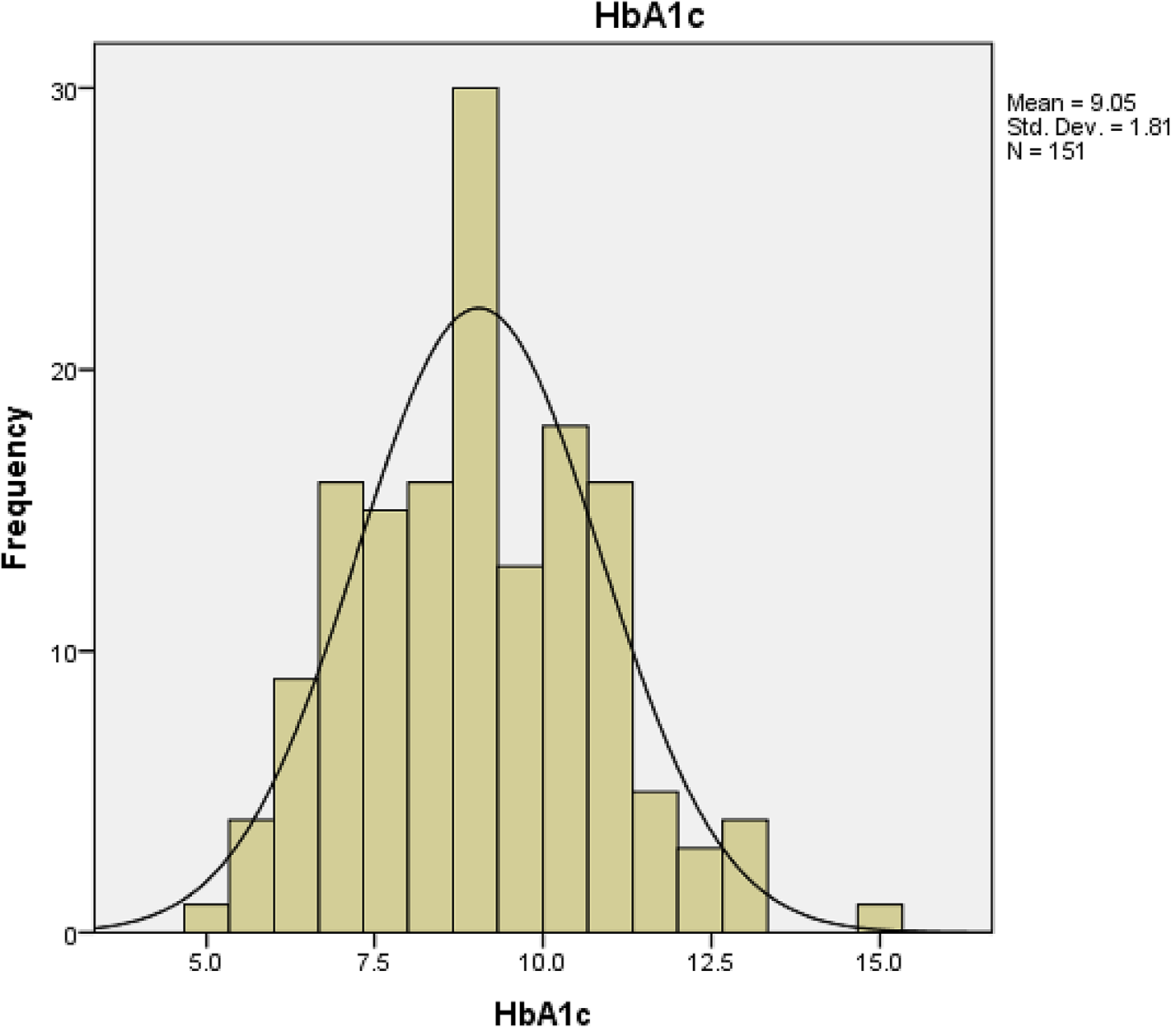
Graphical representation of HbA1c data in the whole cohort.

151/362 were diabetic (42%), and the mean HbA1c was 9% (Figure 3), which highlights poor control among the study group.

Of the cohort, 37% were hypertensive, and 47% were smokers. Only 15 men (4%) gave a positive history of treatment for major CVD and because of the very small sample were excluded from this analysis.

**FIGURE 4:**
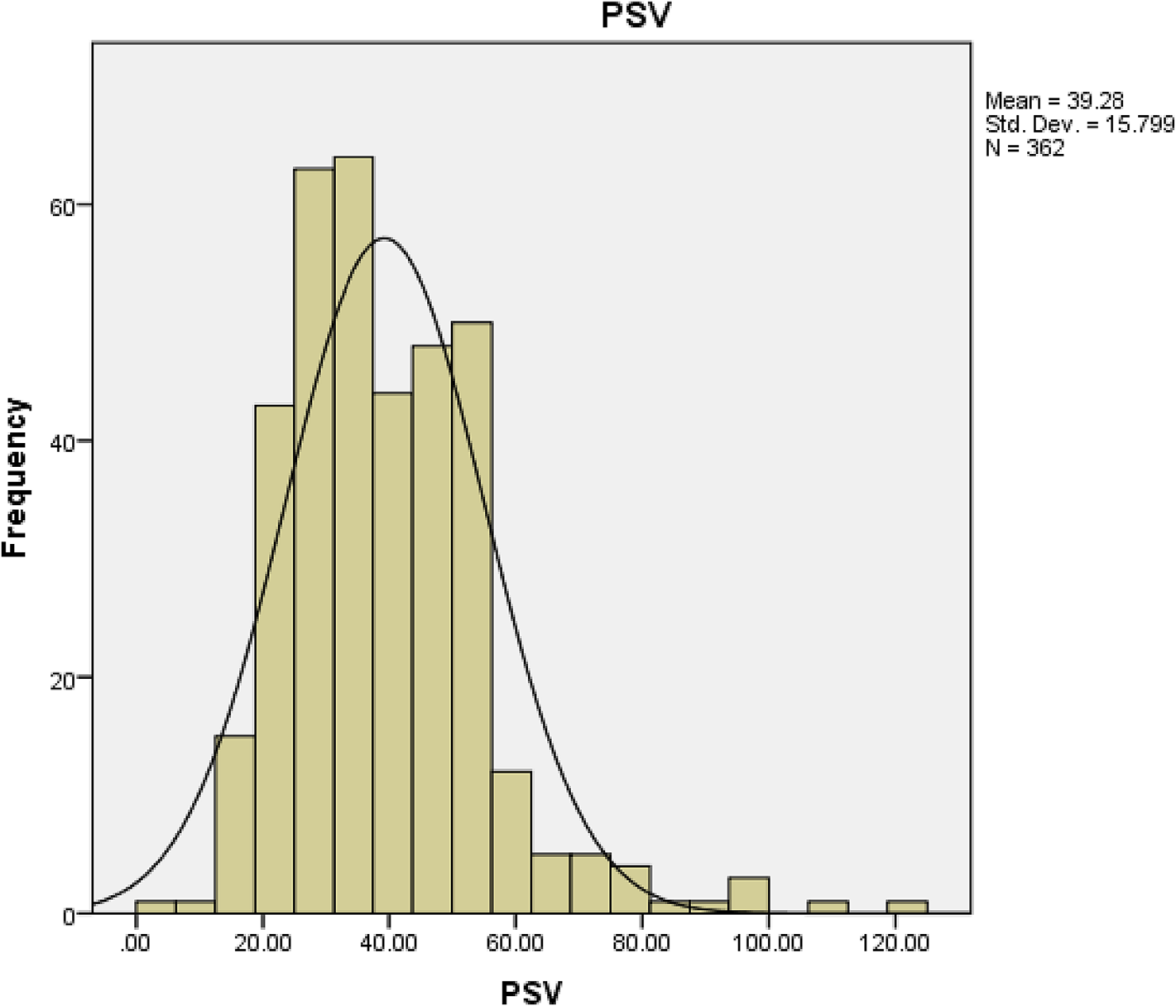
Graphical representation of peak systolic velocity data in the whole cohort.

The mean PSV for the whole cohort was 39.28 cm/s (Figure 4), while the mean EDV was 2 cm/s (0–18).

### 3.1 Age

Older men had lower PSV when compared with younger age, and this correlation was continuous rather than comparing different age groups. This correlation was highly statistically significant (*r* = −0.361, *P* = 0.000). Also, older patients had higher EDV compared to younger men, and this correlation was found to be highly statistically significant (*r* = 0.174, *P* = 0.001).

### 3.2 DM

When comparing diabetics with non‐diabetics, we found that diabetics had lower PSV, and this correlation was found to be statistically significant (*r* = −0.318, *P* = 0.000). Also, for EDV, diabetics were found to have higher EDV than non‐diabetics; this was statistically significant (*r* = 0.139, *P* = 0.008). There was no correlation between HbA1c and PSV (*r* = 0.044, *P* = 0.588).

### 3.3 Hypertension

When comparing hypertensive men with normotensive men, hypertensive men were found to have lower PSV, and this correlation was found to be highly statistically significant (*r* = −0.296, *P* = 0.000). For EDV, there was no correlation.

### 3.4 Smoking

Comparing smokers to non‐smokers, smokers were found to have lower PSV (*r* = −0.140, *P* = 0.008) and higher EDV (*r* = 0.178, *P* = 0.001), and both correlations were highly statistically significant.

### 3.5 BMI

Repeating with an expanded dataset including men with complete PSV data and complete BMI, there were 430 men; there was still no significant correlation between BMI and PSV (0.16, *P* = 0.745).

### 3.6 Dyslipidaemia

There was no statistically significant correlation between dyslipidaemia and PSV or EDV.

### 3.7 Penile size

The mean erected penile length STT was 12.41 cm (7.5–18), while the mean BTT was 14.28 cm (9–20). The mean circumference at the base of the erected penis of the entire cohort was 11.36 cm (5–16).

Men with larger penises measured skin to tip had lower EDV, which was not statistically significant (*r* = −0.119, *P* = 0.024). There were no statistically significant correlations between penile measurements and PSV.

### 3.8 PD

2.5% of participants had PD either confirmed beforehand or as part of investigations into their ED. There was no statistically significant correlations between PD and PSV or EDV.

### 3.9 Testosterone

**FIGURE 5:**
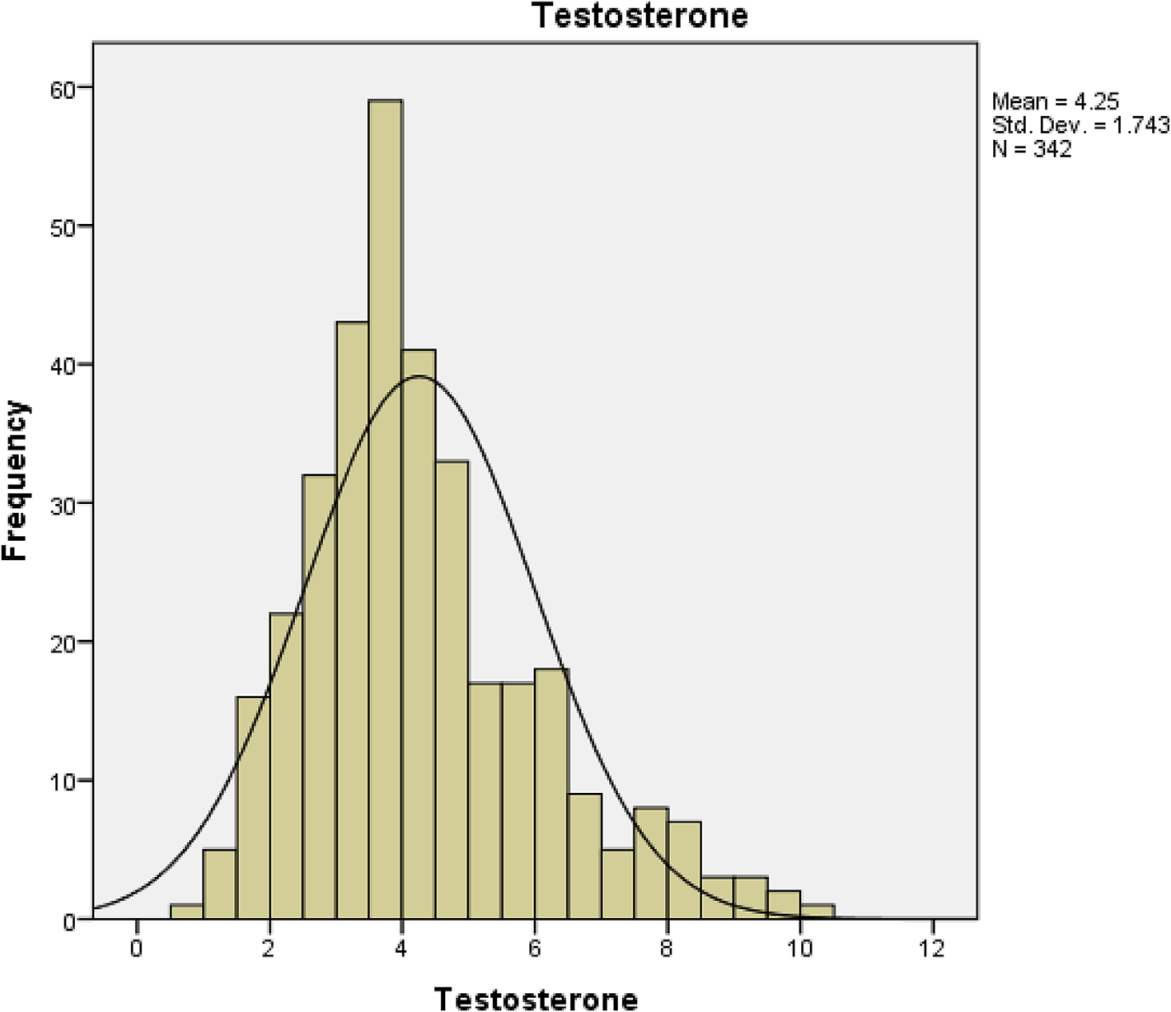
Graphical representation of testosterone data in the whole cohort.

The mean testosterone was 4.25 ng/dL (for more details, see Figure 5). There were no statistically significant correlations between testosterone and PSV or EDV.

**FIGURE 6:**
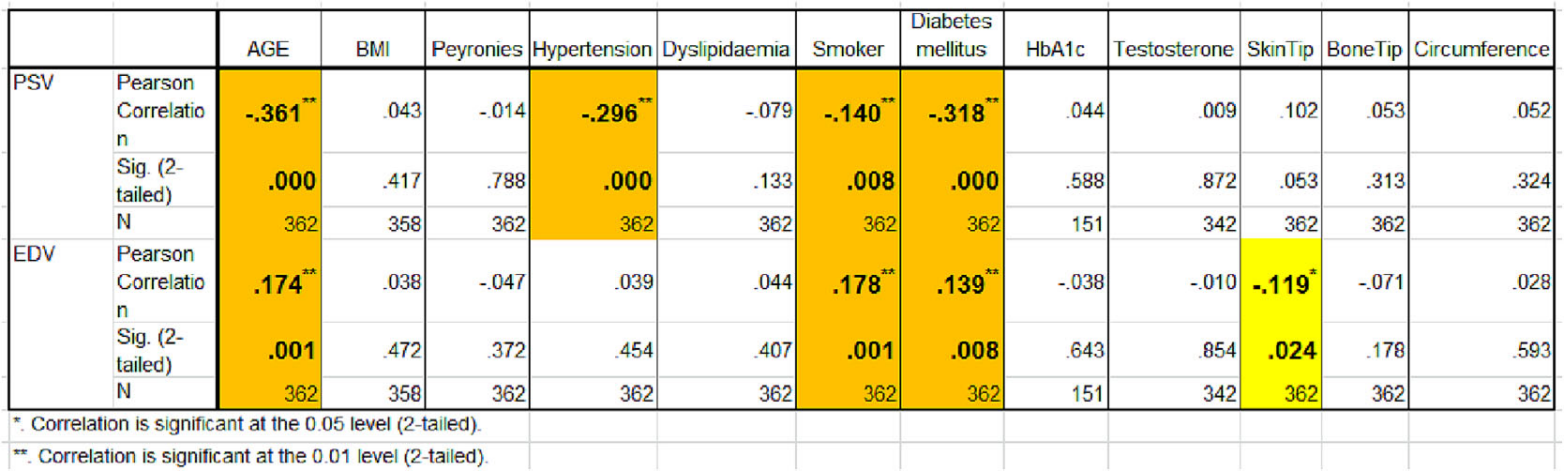
Summary of results and all correlations.

For summary of all correlations, see Figure 6.

## 4 DISCUSSION

ED is a common problem in men above the age of 40, and it has a variety of causes. There are a variety of causes that are often modifiable/treatable. ED has long been seen to have an association with CVD, diabetes and age. Penile cavernosal arteries and coronary arteries are similar in size and calibre, and it stands to reason that disease processes affecting one will influence the other; indeed, published data showed that around half of men with coronary artery disease have ED.^12^ In fact, numerous studies have highlighted the under‐utilisation of ED as an important sign of cardiovascular disease.^12^, ^13^, ^14^, ^15^

In this study, we sought to illustrate the strength of the relationship between presumed vascular risk factors and penile haemodynamics as a surrogate of ED via the use of PDU. Non‐modifiable risk factors we studied were age penile size and disease. Modifiable risk‐factors assessed were Hba1c, dyslipidaemia, hypertension, smoking status, BMI and testosterone.

We found statistically significant correlation between age and reduced PSV as well as higher EDV. This is a well‐established relationship. ED is a major health issue with a high prevalence; it is strongly associated with age and has multiple determinants that all contribute to vascular disease as part of the para‐ageing phenomena.^16^ Patients are living longer and remaining active later in life, and consequently, they have increased expectations towards their erectile function. It is becoming increasingly incumbent upon health professionals to reduce this health complaint via preventative advice and medication where indicated.

Beyond age, diabetics had lower PSV and higher EDV as compared to their non‐diabetic counterparts. This correlates with the known effects of diabetes on endothelial, vascular smooth muscle and platelet function increasing the risk of thrombosis and predisposing to atherosclerosis. Hyperglycaemia, insulin resistance and increased circulating fatty acids, which are inherent in diabetes, promote oxidative stress and decreased bioavailability of nitric oxide (which protects the vessel wall from endogenous injury—atherosclerosis). While HbA1c on its own did not correlate with any statistically significant change in PSV and EDV, the chronicity of poor HbA1c control and an official diabetic diagnosis versus earlier manifestation of elevated HbA1C only with no associated symptomology would account for the difference in this result.^17^ Diabetic control is key for protecting erectile function.

Smokers were also found to have statistically significant poor PDU results. This corroborates the findings of other investigators and highlights smoking cessation as key advice to preserve and improve erectile function.^18^ Smoking, much like diabetes, increases the oxidative stress on vascular walls predisposing to atherosclerosis and through sympathetic nervous activation causes transient hypertension while increasing the chance of chronic hypertension.^19^ Hypertension as a risk factor in itself did illustrate poorer PSV with no effect demonstrated on EDV, which correlates with physiological expectation. The effect of hypertension on ED can potentially be a consequence of the blood pressure itself or the antihypertensive agent used in treatment. Newer agents tend to be better tolerated, and again, clinicians need to appreciate the effect of both when treating ED.

We had hypothesised that longer penises may need a higher PSV to maintain erection, but found no evidence of that in this cohort—it is possible that we may see a different result if including a larger number of very long penises.

With regard to BMI, while it would be expected that high BMIs (>25) would correlate with poorer penile haemodynamics, we did not demonstrate this in our study. It should be noted that BMI does not necessarily relate to the haematological lipid profile of patients. Beyond that, the venous congestion caused by obesity may contribute to higher EDV. Long‐term however, BMI and vascular disease are intrinsically linked; therefore, maintaining a healthy weight is important in lasting erectile function.

PD and testosterone had no effect on PDU results, neither did penile size. We had relatively few participants with PD, and thus, we cannot draw conclusions from this. Testosterone levels have historically been shown to have less impact on ED than CVS disease; this is in keeping with the fact that erections are predominantly a vascular process in its machinations.^20^ Larger penile sizes did not result in poorer PDU, and anatomically speaking generally, a larger penis will be served by larger arteries, therefore maintaining normal PDU values.

We agree that PDDU is an operator‐dependent procedure, but it remains the best option to diagnose vascular causes of ED, being less invasive and safe (no hazards of radiation or hypersensitivity reactions as in cavernosography). It is also cheap and easy test available in almost every facility worldwide.

Our study was adequately powered, with robust and reproducible techniques to perform PDU. The study was not blinded, but reporting and measurement bias was reduced by using the same equipment and staff throughout. However, the exclusion criteria did include patients who did not respond to ICI stimulation, thus potentially skewing results.

## 5 CONCLUSIONS

Our study highlights key risk factors—increased age, tobacco consumption, DM and hypertension—which have a statistically significant negative impact on penile haemodynamics. Of these, tobacco consumption, good diabetic control and hypertension are potentially modifiable and should form part of any treatment plan for erectile dysfunction.

Other risk factors—increased BMI, low testosterone, PD and penile size—had no statistically significant impact on penile haemodynamics. This allows for targeted advice that can be given to patients who present with this very common problem. Ultimately, good vascular health goes hand in hand with good erectile function.

## AUTHOR CONTRIBUTIONS

*Conceptualization*: Mohamad Habous and Gordon Muir. *Methodology*: Mohamad Habous, Mohammed Aziz, Mohammed Farag, and Saleh Binsaleh. *Statistical analysis*: Ahmed Khattak. *Software*: Mohamad Habous and Saleh Binsaleh. *Validation*: Mohamad Habous and David Ralph. *Formal analysis*: Gordon Muir and Ahmed Khattak. *Investigation* and *resources*: Mohamad Habous and Carlo Bettocchi. *Data curation*: Mohamad Habous, Gordon Muir, Mohammed Farag, and Saleh Binsaleh. *Writing—original draft*: Mohamad Habous, Mohammed Aziz, Gordon Muir, and Ahmed Khattak. *Review and editing*: Mohamad Habous, David Ralph, and Carlo Bettocchi. *Visualization and supervision*: Mohamad Habous. *Project administration*: Mohamad Habous. *Funding acquisition*: Mohamad Habous.

## CONFLICT OF INTEREST STATEMENT

None.
